# Integrated Nutrient Management Improves the Growth and Yield of Rice and Greengram in a Rice—Greengram Cropping System under the Coastal Plain Agro-Climatic Condition

**DOI:** 10.3390/plants11010142

**Published:** 2022-01-05

**Authors:** Satyabrata Mangaraj, Rabindra Kumar Paikaray, Sagar Maitra, Shriram Ratan Pradhan, Lalita Mohan Garnayak, Manoranjan Satapathy, Barsita Swain, Satyananda Jena, Bijayalaxmi Nayak, Tanmoy Shankar, Mohammed Alorabi, Ahmed Gaber, Akbar Hossain

**Affiliations:** 1 Department of Agronomy, Odisha University of Agriculture and Technology, Bhubaneswar 751003, Odisha, India; Satyabratamangaraj7@gmail.com (S.M.); rkpaikaray@rediffmail.com (R.K.P.); lmgarnayak@yahoo.co.in (L.M.G.); mrsouat@gmail.com (M.S.); sbarsita96@gmail.com (B.S.); snjena2008@gmail.com (S.J.); 2 Department of Agronomy, Centurion University of Technology and Management, Paralakhemundi 761211, Odisha, India; sagar.maitra@cutm.ac.in (S.M.); tanmoy@cutm.ac.in (T.S.); 3 Department of Vegetable Science, Odisha University of Agriculture and Technology, Bhubaneswar 751003, Odisha, India; ramsushree@gmail.com; 4 Department of Extension Education, Sikhsha ‘O’ Anusandhan University, Bhubaneswar 751030, Odisha, India; nbijayalaxmi1992@gmail.com; 5 Department of Biotechnology, College of Science, Taif University, P.O. Box 11099, Taif 21944, Saudi Arabia; maorabi@tu.edu.sa; 6 Department of Biology, College of Science, Taif University, P.O. Box 11099, Taif 21944, Saudi Arabia; 7 Department of Agronomy, Bangladesh Wheat and Maize Research Institute, Dinajpur 5200, Bangladesh

**Keywords:** short grain aromatic rice, integrated nutrient management, cropping system, greengram, productivity, nutrient balance

## Abstract

Continuous mono-cropping of rice has resulted in decline or stagnation of yield output due to the occurrence of multiple nutrient deficiencies and worsening of soil physicochemical properties accompanying increased pressure of insect pests and diseases. The basic concept of integrated nutrient management (INM) is maintenance or adjustment of soil fertility and supply of plant nutrients to an optimum level for sustaining the desired crop productivity through optimisation of benefits from all possible sources of plant nutrients in an integrated way. Augmenting a rice-based cropping system with pulses is a prevalent and indigenous cropping system under rainfed conditions. Considering the above facts, experiments were conducted to evaluate the impacts of integrated nutrient management on productivity of aromatic rice–greengram cropping system and nutrient balance of the post-harvest soil for agricultural sustainability under rainfed conditions in two consecutive years (2017–2018 and 2018–2019) with six main plots and three subplots. The experimental findings revealed that the treatment comprised of 50% recommended dose of fertiliser (RDF) through chemicals + 50% recommended dose of nitrogen (RDN) through farmyard manure (FYM) increased the plant height, tillers, dry matter accumulation, leaf area and leaf area duration, and yield parameters in short grain aromatic rice. Similarly, preceding application of 50% RDF + 50% RDN through FYM to rice and further application 75% RDF + *Rhizobium*+ phosphate solubilizing bacteria (PSB) to greengram increased the growth characteristics and yield parameters—such as pods/plant, seeds/pod, grain yield, stover yield, and harvest index—in greengram. It was concluded that the treatment consisting of 50% RDF (chemical fertiliser) + 50% RDN (FYM) to rice and 75% RDF + *Rhizobium* + PSB to greengram increased the productivity of the rice–greengram cropping system. Furthermore, the adoption of INM has positively impacted post-harvest soil nutrient balance.

## 1. Introduction

Rice (*Oryza sativa* L.) is the most important and widely grown food grain in the world. It is accredited as a ‘global grain’ because of its elementary role as a staple food all around. Indeed, rice has yielded itself into our lives and hearts [1]. India has rich genetic diversity for landraces of aromatic rice; of them, major landraces are of small-to-medium grains which are classified into a separate group [2,3]. The indigenous short grain aromatic rice cultivars are cultivated in limited areas in various corners of the country. These rice cultivars contain vital natural chemical compounds, such as 2-acetyl-1-pyrroline, with essential amino acids—particularly lysine, leucine, phenylalanine, and methionine—and these cultivars have excellent sensory characteristics with local demand as a delicacy [3]. Furthermore, these short grain aromatic rice cultivars have an edge over traditional basmati rice because of the varied and intense aroma with long retention capacity in a relatively warmer region. During recent times, the requirement of aromatic rice has augmented to a greater extent for both domestic and export markets. Peculiar grain quality, appealing aromatic flavour, higher nutrient standards, and considerable auspiciousness have added up to the prime position of aromatic rice in the domestic circuit and international market. Most of the import and export of aromatic rice in the world is also from India, Pakistan, and Thailand. Consumers have become more quality conversant. Therefore, quality parameters are also considered equally along with enhancement of production and productivity. When farmers become conscious of their rice quality, they are motivated to produce superior quality rice [4,5].

The farmers have switched over to high-yielding modern varieties because of the higher yield which compensates for the premium price of aromatic rice. Traditional aromatic rice varieties are tall, susceptible to disease and pest incidence, and are low yielders. The yield from conventional varieties is still low compared to its potential which needs to be boosted while maintaining or improving the quality with efficient utilisation of input factors.

Rice-based cropping systems are being scrutinised for their questionable sustainability. With the heavy addition of fertilisers and agrochemicals, rice soils are getting deprived of their inherent fertility which is a potential concern for soil health in the long run. The escalating costs of these chemicals have also raised eyebrows. These challenges have renewed interest in organic alternatives. Chemical nutrients are well recognised for their effective yield increase, while organic inputs improve the aroma as well as quality [6]. To feed the rising population, the production and productivity of crops are to be intensive. This demand cannot be met by the sole use of chemical or organic nutrient inputs for plants; rather, a combination of both is more practical, economical, and viable for the producers, end-users, and soil environment [7,8]. A judicious and rational fertiliser usage can advertently enhance productivity and improve rice quality [9,10].

Inclusion of green manure, farmyard manure (FYM), and biofertilisers—such as *Azospirillum*, *Rhizobium*, and PSB—help in supplementing crop requirements and reforming the physicochemical and biological properties of soil in the cropping system [11,12]. Biofertilisers are cheap, eco-friendly, and provide nutrients to the crop for a prolonged period. Farmyard manure and green manure act as soil conditioners by providing a congenial environment for the growth of the microbial population. Organic sources, apart from improving intrinsic properties of soil, help in enhancing the use efficiency of fertilisers. Integrating mineral fertilisers with organic manures offer an environmentally safe, economically sound, socially reasonable, and ecologically sustainable production system [13,14]. Amongst the essential elements, nitrogen has a prominent role in the growth and metabolic processes in rice, consequently contributing to more than 50% of the yield increment, even under usual growing conditions. 

Strategic incorporation of recommended fertiliser dosing in addition to locally available organic manures should be employed to satisfy the nutrient requirement of crops for higher yield without impairing soil health with optimum input levels. The residual effect of organic manure has proven adequate for subsequent crops. In addition to that, these organic substitutes proliferate the soil microbial status. Comprehending soil microbial ecology is increasingly acknowledged as the principal key for the restoration and sustainability of the soil ecosystem. The amount of soil microbes’ diversity in farmland is crucial for maintaining soil health and quality. The improved microbial activities not only enhance the nutrient transformation rate, forming readily available plant nutrients, but also have a positive effect on crop growth because of enzymatic activities and suppression of diseases [15].

With the advantage of the considerable area under rice fallow and retreating monsoon, pulse crops (such as greengram) fit into the system and are preferred by the farming community due to their low nutrient requisites, short duration, and beneficial effects thereby [16]. In addition to being an important source of human food and animal feed, an important feature of the greengram crop is its ability to establish a symbiotic relationship with specific bacteria, setting up the biological nitrogen fixation in root nodules that supply the plants’ needs for nitrogen. The green biomass of the crop, as well as residues, can be incorporated into the soil to replenish exported plant nutrients and improve the fertility status of the soil. The soil microbiological properties were also significantly higher in soils where mung bean is incorporated in the cropping system. Despite the studies on nutrient utilisation in the cropping systems of different crops, considerable work is still needed on the residual impact of various nutrient management practices adopted in rice on the performance of succeeding greengram concerning growth and productivity. Considering the above facts, this experiment was conducted to evaluate the impacts of integrated nutrient management on productivity of an aromatic rice–greengram cropping system and nutrient balance of the post-harvest soil for agricultural sustainability.

## 2. Materials and Methods

### 2.1. Site Description 

The experiment was conducted during rainy and winter seasons in 2017–2018 and 2018–2019 at Instructional Farm of Department of Agronomy, College of Agriculture, Odisha University of Agriculture and Technology, Bhubaneswar, India (20°15′ N, 85°52′ E, 25.9 m above mean sea level, Odisha, India). The station lies within the east and south-eastern coastal plain agro-climatic zone of Odisha, India. Representative soil samples were collected from a depth of 15 cm in a zig-zag manner before land preparation to analyse the textural class and inherent physicochemical status. The soil was sandy loam having good water holding capacity and internal drainage. The soil characteristics were slightly acidic, low in organic carbon and available nitrogen, and medium in available phosphorus and available potassium. Data on the chemical analysis of experimental soil (0–15 cm depth) at the experimental site is presented in Table 1.

### 2.2. Meteorological Conditions 

**Figure 1:**
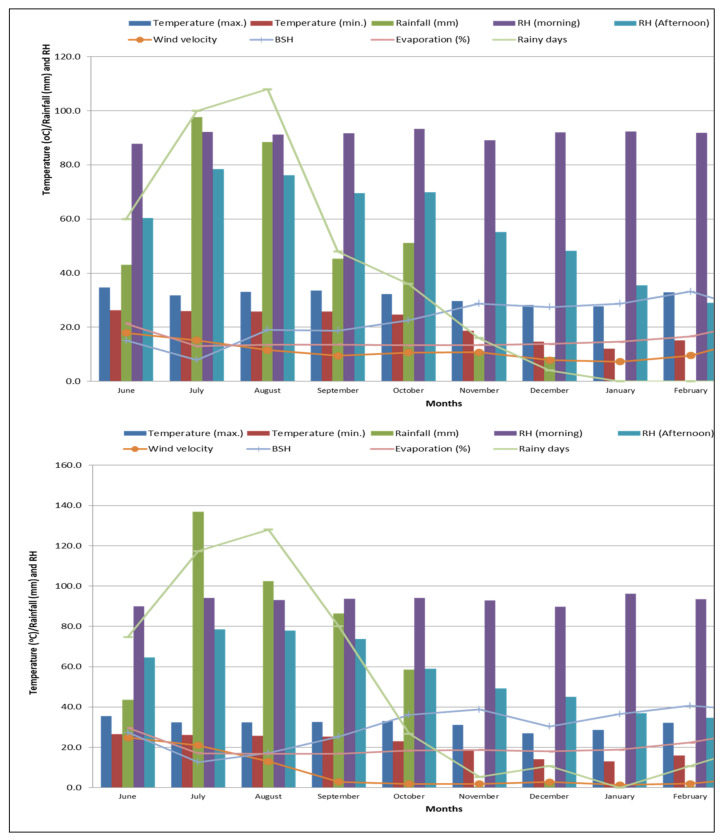
Month wise meteorological data during the crop growing seasons in 2017–2018 and 2018–2019 (Department of Agrometeorology, College of Agriculture, OUAT, Bhubaneswar, Odisha, India).

A detailed of weather parameters in both growing seasons of the current study are given in Figure 1.

The climate is warm and moist with a hot and humid summer and mild winter. Rainfall received during June to November in 2017 and 2018 was 1446.8 mm (88 rainy days) and 1211.1 mm (80 rainy days), respectively, which was sufficient for rice cultivation under rainfed conditions (Department of Agrometeorology, College of Agriculture, OUAT, Bhubaneswar, Odisha, India). The subsequent crop of greengram received 91.5 mm of rainfall for a total number of 5 rainy days in the cropping season of 2017–2018 and 35.7 mm rainfall in 5 rainy days in 2018–2019. Maximum temperature (39.5 °C) was witnessed in June 2017 whereas minimum temperature (11.0 °C) was experienced in January 2018. Similarly, the maximum temperature of 38.3 °C was noted in June 2018 and the minimum temperature of 10.2 °C was observed in January 2019. 

### 2.3. Treatments and Layout

The experiment was laid out in a split-plot design with three replications. Six nutrient management practices in rice were allotted as the main plots in Kharif, i.e., G_1_: 100% recommended dose of fertiliser (60-30-30 N-P_2_O_5_-K_2_O kg/ha), G_2_: 75% recommended dose of fertiliser (RDF)+ 25% recommended dose of nitrogen (RDN) through farmyard manure (FYM), G_3_: 50% RDF + 50% RDN through FYM, G_4_: 50% RDF + 25% RDN through FYM, G_5_:75% RDF + green manuring and G_6_: 50% RDF + green manuring. Organic manures (FYM and *dhaincha*, as green manure grown prior to rice and green plants incorporated into the soil) were applied to rice based on the treatments. *Azospirillum* and phosphate solubilizing bacteria (PSB) were in all treatments. Three nutrient management practices in greengram as the sub-plots—i.e., P_1_: 100% RDF (20-40-40 N-P_2_O_5_-K_2_O kg/ha)—P_2_: 75% RDF and P_3_: 75% RDF + *Rhizobium* + PSB were laid out in rabi season. The experiment was started in 2017–18 with short grain aromatic rice as Kharif season crop followed by greengram as the rabi season crop. In the Kharif season, the main plot treatments were allotted randomly to different experimental units of rice and in the rabi season, the subplot treatments to greengram were allotted randomly within main plots of rice (Kharif aromatic rice).

### 2.4. Crop Culture

Seedlings of short grain aromatic rice variety “Nua Acharamti”, a high yielding variety with local demand, were transplanted with a row × plant spacing of 20 × 15 cm, keeping 2–3 seedlings hill^−1^ during Kharif. *Dhaincha (Sesbania aculeata*) at 25 kg/ha was seeded on the onset of monsoon in G_5_ and G_6_ and incorporated at 40–42 days after sowing (DAS). Before the incorporation of organic manures, NPK contents were analysed and nutrient content of organic manures used in the experiment are given in Table 2. 

PSB and *Azospirillum* were applied to all six treatments. After the harvest of Kharif aromatic rice, minimum soil was disturbed while preparing land for *rabi* greengram. The fixed plots were cultivated with a power tiller. Lines were drawn manually in each plot keeping a space of 30 cm within rows. All the inorganic were applied as per treatments at the time of sowing. The required quantity of *Rhizobium* cultures, i.e., at 200 g culture/10 kg seed was mixed to 10% sugar solution to form a slurry. The culture of PSB 200 g/12 kg fine soil was well mixed and applied to the field as per treatment details. Seeds of greengram cv. IPM-02–03 were used for sowing. In rice, for weed management, the pre-emergence herbicide used as pretilachlor 6% + bensulfuron methyl 0.6% at the rate of 600 g per ha along with sand and applied on 3 days after transplanting (DAT). Subsequently, one hand weeding was carried out at 45 DAT. In the case of greengram, pre-emergence application of pendimethalin at the rate of 1 kg per ha was done at three DAS followed by one hand weeding at 35 DAS. The same weed management practices were carried out for the crops during both years. For pest management in rice, neem seed kernel extract at 3% and fipronil 5% SC (at 1.0 mL/L of water) were sprayed twice against the sucking pest of rice in the first year and in the second year no pesticide was applied. However, greengram did not face any attacks from pests and diseases during both years and pesticides were not applied.

### 2.5. Plant Sampling

Periodical observations were recorded for both crops. From the five tagged plants of rice, plant height was recorded from the ground to the tip of the topmost leaf at 30-day intervals up to harvest. During maturity, the height was recorded from the ground to the tip of the panicle. The tillers of rice from the one-metre square were counted in the field at successive intervals of 30 days up to 90 DAT and averaged out to find out tiller number m^−2^. However, at maturity total tillers along with panicle bearing tillers were counted and recorded as ear bearing tillers. The culms, leaves, and panicles (after panicle emergence) of the above mentioned collected samples were separated, air-dried in the shade, followed by drying in the hot air oven at 70 °C till a constant weight was obtained. The net area of individual plots was harvested manually. The produce was sundried. Then the bundles were threshed plot-wise. The grains were winnowed separately, then cleaned and sundried for 4 days. The weights of grain, straw, and chaff were noted separately. The moisture content in the grain was determined. The grain and straw yield were finally converted in kg/hectare at 14% moisture content. Height and branches of the five selected tagged plants of greengram in each plot were recorded at 30 DAS, followed by 15-day intervals up to maturity. The plot-wise seed yield was noted after drying the seed under the sun to standard moisture content.

### 2.6. Statistical Analysis

All the biometric data recorded were compiled in tables and subjected to statistical analysis as per the procedure prescribed for randomised block design (RBD) and split-plot design (SPD) to obtain the analysis of the variance. The treatment variations were tested for significance by ‘F’ test. The standard error of mean, SE (m) ±, and least significance difference (LSD) at 5% probability level was calculated as per the formulae mentioned by Gomez and Gomez [22] to interpret the result. All the data were subjected to Tukey’s HSD test.

## 3. Results

### 3.1. Growth Parameters of Short Grain Aromatic Rice as Influenced by Integrated Nutrient Management

The plant height increased successively with advancement in crop growth stages until harvest. The rate of enhancement in plant height was maximum during 30–60 DAT which coincided with the elongation stage of crops and it reached near plateau at the time of maturity (Table 3). The tallest plant during different growth stages was registered with 50% RDF (chemical) + 50% RDN (FYM) in both years. Significantly, taller plants (135.4 and 138.1 cm) were recorded with 50% RDF + 50% RDN through FYM over other treatments and closely followed by the treatment consisting of 75% RDF + green manuring (127.9 and 131.4 cm) and 75% RDF + 25% RDN through FYM (122.8 and 124.0 cm) at harvest. The shortest plants (111.9 and 111.0 cm) at harvest were found under the treatment receiving 50% RDF + 25% RDN through FYM and also followed the same trend in other growth stages (Table 3).

Tillers/m^2^ at all growth stages was significantly influenced by integrated nutrient management practices in rice (Table 4). There is a rapid increase in tiller number from 30 DAT to 60 DAT with a little increase from 60 DAT to 90 DAT during both years of the experiment. The treatment consisting of 50% RDF + 50% RDN (FYM) noted the maximum tillers/m^2^ (359.3 and 372.6) at 90 DAT closely followed by application of 75% RDF + green manuring (341.9 and 351.0) at 90 DAT. The most inferior tillers/m^2^ was observed under the 50% RDF + 25% RDN through FYM at all growth stages (Table 4).

**Figure 2:**
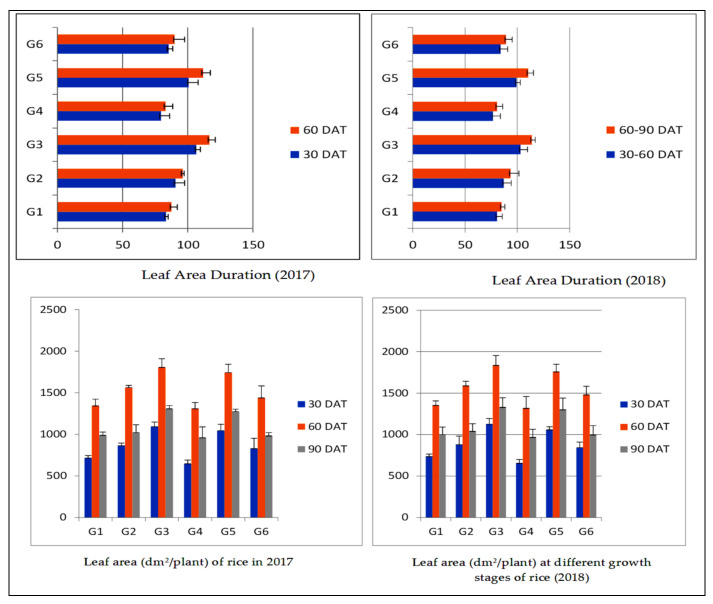
Leaf area duration and leaf area of short grain aromatic rice influenced by integrated nutrient management practices. For treatment details, Table 2 may be referred. SE, standard errors; LSD, least significance difference at 5% probability level.

Leaf area and leaf area duration of short grain aromatic rice varied significantly with integrated nutrient management practices and given the highest values in 50% RDF + 50% RDN (FYM) being at par with 75% RDF + green manuring (Figure 2). The total dry matter of aromatic rice enhanced with the progression of the crop age and touched the highest at harvest (Table 5). The enhancement rate was slower during the first 30 DAT but got increment after 60 DAT due to the formation of reproductive parts. The rate of increase was maximum during 60–90 DAT. The treatment consisting of 50% RDF + 50% RDN (FYM) increased total dry matter at 30, 60, and 90 DAT, respectively. At harvest, significantly maximum dry matters (770.4 and 757.1 g/m^2^) were obtained due to the application of 50% RDF + 50% RDN through FYM being at par with the application of 75% +green manuring (743.9 and 757.7 g/m^2^). The lowest values were obtained by application of 50% RDF + 25% RDN through FYM during different growth stages in the study period (Table 5). Leaf area duration (LAD) of aromatic rice increased from 30–60 DAT to 60–90 in all treatments applied. Significantly higher LAD (105.0 and 115.3 days) at 30–60 and 60–90 DAT were obtained with 50% RDF + 50% RDN (FYM) closely followed by 75% RDF + green manuring (100.2 and 111.3 days in 2017 and 2018, respectively). The least values (78.3 and 81.9 days) were observed in 50% RDF + 25% RDN through FYM treatment (Figure 2).

### 3.2. Yield Parameters of Short Grain Aromatic Rice as Influenced by Integrated Nutrient Management

Yield parameters of rice were influenced by different integrated nutrient management practices to short grain aromatic rice (Table 6). Among different treatments, the significantly higher ear bearing tillers/m^2^ (315.7 and 321.0) were registered with 50% RDF + 50% RDN through FYM over other treatments in 2017 and 2018 being at par with 75% RDF + green manuring (305.4 and 312.6) and 75% RDF + 25% RDN through FYM (295.8 and 302.6).

Application of 50% RDF + 50% RDN (FYM) resulted in significantly more filled grains/panicle (178.6 and 182.6) in 2017 and 2018 but the treatment received 50% RDF + 25% RDN through FYM produced the lowest values (125.2 and 132.5, respectively). The filled grains/panicle in 50% RDF + 50% RDN through FYM remained statistically at par with the application of 75% RDF + green manuring (160.2 and 164.2).

The treatment consisting of 50% RDF + 50% RDN through FYM significantly registered the highest length of panicle (27.6 and 27.9) being at par with the treatment receiving 75% RDF + green manuring (27.0 and 26.9), 75% RDF + 25% RDN through FYM (25.1 and 25.9), and 50% RDF + green manuring (24.2 and 25.5) in 2017 and 2018, respectively. Application of 50% RDF + green manuring obtained the least length of panicle (20.0 and 20.6).

The treatment receiving 50% RDF + 50% RDN through FYM recorded significantly higher test weight (18.2 and 18.6 g) being at par with 75% RDF + green manuring. Further results showed that application of 75% RDF + 25% RDN through FYM, 50% RDF + green manuring, and 100% RDF were at par with each other. The lowest values were obtained by application of 50% RDF + 25% RDN through FYM in both years.

The results showed that different treatments had a significant impact on grain yield. The treatment receiving 50% RDF + 50% RDN through FYM recorded significantly highest grain yield (3837 and 3914 kg/ha) over other treatments being at par with 75% RDF + green manuring (3438 and 3539 kg/ha) in 2017 and 2018. Furthermore, results showed that grain yield from the treatment received 75% RDF + 25% RDN through FYM (3334 and 3328 kg/ha), 50% RDF + green manuring (3182 and 3224 kg/ha) were at par with each other. The most inferior values were obtained with 50% RDF + 25% RDN through FYM during both years of study.

**Figure 3:**
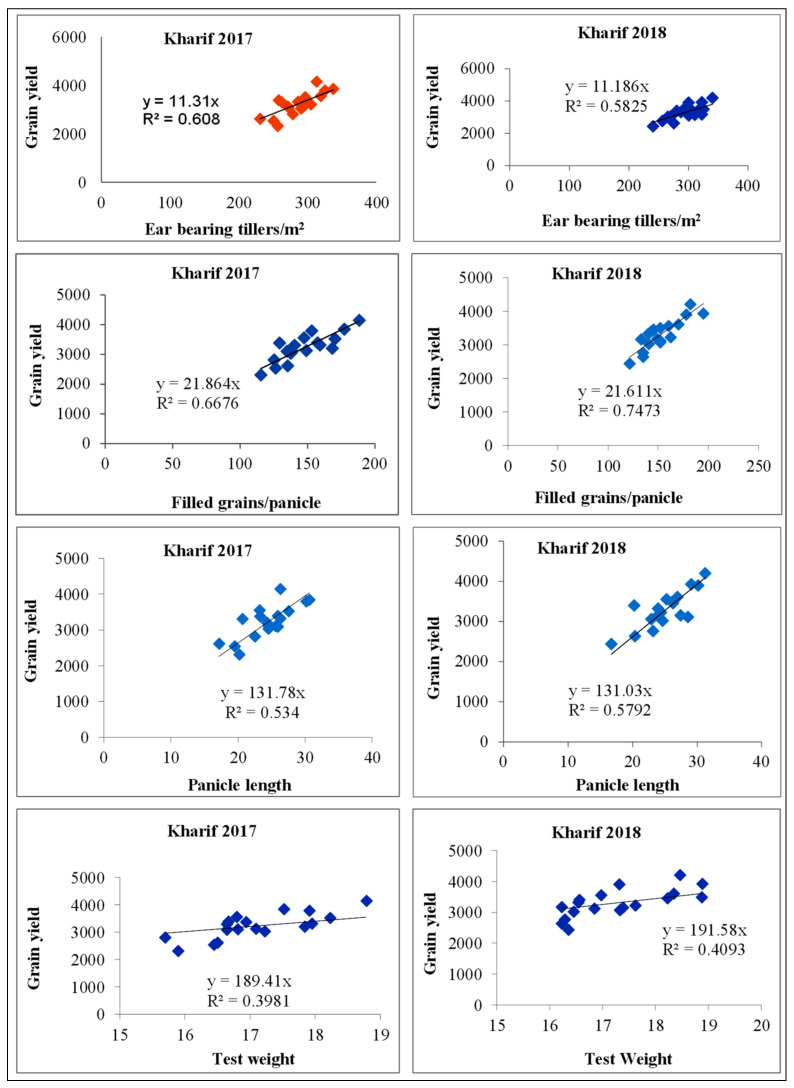
Correlation between yield parameters and grain yield of short grain aromatic rice.

Significantly higher straw yield (5278, 5342 kg/ha) in 2017 and 2018 was found by the treatment comprised of 50% RDF + 50% RDN (FYM) over other treatments being at par with 75% RDF + green manuring (4924 and 4931 kg/ha). Further results showed that the treatments receiving 75% RDF + 25% RDN through FYM and 50% RDF + green manuring were at par with each other. The lowest values were obtained from 50% RDF +25% RDN through FYM in both the years of study. Among different INM practices followed, a significantly higher harvest index (42.1 and 42.2%) was recorded with the treatment consisting of 50% RDF +50% RDN through FYM being at par with the treatment comprised of 75% RDF + green manuring in 2017 and 2018. The lowest straw yield was obtained from 50% RDF + 25% RDN through FYM (37.5 and 37.4) (Table 6). Correlation studies revealed that a positive and strong correlation existed between grain yield and yield parameters of rice like filled grains/m^2^, ear bearing tillers, panicle length, and positive correlation between grain yield and test weight (Table 7 and Figure 3).

### 3.3. Growth Parameters of Greengram

The average plant height of greengram was enhanced with an increase in progression of crop age and reached its peak at harvest. With respect to nutrient management practices applied to preceding aromatic rice, application of 50% RDF +50% RDN through FYM resulted in significantly higher plant height over other treatments at all growth stages and harvest highest plant height (37.3 cm) was recorded as being at par with 75% RDF + green manuring (36.4 cm). Furthermore, results revealed that 75% RDF + 25% RDN through FYM and 50% RDF + green manure were at par with each other. The lowest values were obtained by application of 50% RDF +25% RDN through FYM to preceding rice. Similarly, a significant difference in plant height was observed by the application of INM practices to *rabi* greengram at all growth stages. Significantly, taller plant height (34.9 and 35.8 cm) was found by application of 75% RDF + *Rhizobium*+ PSB which was 3.8 and 11.3% higher than 100% RDF and 75% significant (Table 8).

Application of nutrient management practices to preceding rice had a significant effect on dry matter accumulation of greengram at all growth stages. Significantly higher dry matter accumulation of greengram was produced by the treatment 50% RDF + 50% RDN through FYM at 30, 45, and 60 DAS. At harvest, significantly higher dry matter accumulation (15.9 and 16.3 g/plant) in 2017–2018 and 2018–2019, respectively were observed by 50% RDF + 50% RDN followed by 75% RDF + green manuring (14.6 and 15.0 g/plant). Lower values were obtained by 50% RDF + 25% RDN through FYM treatment (8.9 and 9.1 g/plant). Similar trends were observed at other growth stages. Similarly, application of 75% + *Rhizobium* + PSB to greengram recorded significantly higher dry matter accumulation (14.2 and 14.6 g/plant) followed by 100% RDF (12.9 and 13.10 g/plant) and 75% RDF (11.7 and 11.9 g/plant) (Table 9).

### 3.4. Yield Parameters of Greengram

Among different INM practices applied to preceding rice, application of 50% RDF + 50% RDN through FYM significantly produced the highest pods/plant (11.0 and 11.1) followed by the treatment receiving 75% RDF + green manuring (10.2 and 10.4) and 75% RDF + 25% RDN through FYM (8.3 and 8.7) as per both years of study (Table 10). 

The lowest values were obtained by 50% RDF + 25% RDN through FYM. Similarly, application of 75% RDF + *Rhizobium* + PSB gave the highest pods/plant (9.2 and 9.6) followed by 100% RDF and 75% RDF. Application of 50% RDF + 50% RDN through FYM recorded significantly higher seeds/pod (10.8), pod length remaining at par with 75% RDF + green manuring (10.7). Furthermore, results revealed that 75% RDF + 25% RDN through FYM and 75% RDF + green manuring were at par with each other. The lowest values were obtained from 50% RDF +25% RDN through FYM during both years of study.

Significantly, the highest seed yield of greengram (798 and 815 kg/ha) in 2017–2018, 2018–2019 was recorded by residual application of 50% RDF +50% RDN through FYM to rice and closely followed by 75% RDF + green manuring (to rice) on individual years of study. 50% RDF +25% RDN through FYM treatment gave the lowest values in the study. Furthermore, the treatment 75% RDF + *Rhizobium* + PSB to greengram gave comparatively higher seed yield (684 and 710 kg/ha) in 2017–2018 and 2018–2019 amongst the treatments provided to greengram—i.e., the second crop grown in the system. The lowest values were obtained from the application of 75% RDF.

With respect to nutrient management practices to preceding rice, application of 50% RDF +50% RDN through FYM obtained higher stover yield (1993 and 1922 kg/ha) in 2017–18, and 2018–2019 respectively, followed by application of 75% RDF + green manuring and 75% RDF + 25% RDN through FYM. Similarly, application of 75% RDF + *Rhizobium*+ PSB to greengram gave the highest stover yield (1784 and 1749 kg/ha) in 2017–2018 and 2018–2019 followed by 100% RDF (1685 and 1603 kg/ha) (Table 10). 

### 3.5. Nutrient Balance in Soil at the End of the Cropping System

#### 3.5.1. Nitrogen Balance in Soil 

Data on nitrogen balance in soil is presented in Table 11. Integrated nutrient management practices to rice–greengram system positively influenced the net balance of nitrogen in soil at the end of the system. Total highest N uptake in the cropping system was obtained from 50% RDF + 50% RDN through FYM treatment (332.0 kg/ha). Application of 75% RDF + green manuring to short grain aromatic rice crop and 75% RDF + *Rhizobium* + PSB in greengram significantly increased available N (225.7 and 217.1 kg/ha, respectively) in soil at the end of the system. Net gain in nitrogen status in soil was maximum by application of 75% RDF + green manuring (26.4 kg/ha) followed by 50% RDF + 50% RDN through FYM (21.9 kg/ha) and 75% RDF + 25% RDN through FYM (19.5 kg/ha). The lowest gain was observed with 50% RDF + 25% RDN through treatment. Similarly, application of 75% RDF + Rhizobium + PSB to greengram produced a positive net gain in nitrogen availability (17.8 kg/ha) followed by 100% RDF (15.7 kg/ha) and 75% RDF (13.8 kg/ha).

#### 3.5.2. Phosphorus Balance in the Soil at the End of Cropping System

Data on phosphorus balance in soil is presented in Table 12. The treatment receiving 50% RDF + 50% RDN through FYM to rice and 75% RDF + *Rhizobium* + PSB to greengram produced significantly higher total phosphorus uptake (57.4 and 46.1 kg/ha, respectively). Net gain in phosphorus status in soil was maximum by application of 75% RDF + green manuring (8.9 kg/ha) followed by 50% RDF + 50% RDN through FYM (7.2 kg/ha) and 75% RDF + 25% RDN through FYM (4.2 kg/ha). Lowest gain was observed with 50% RDF + 25% RDN through treatment. Similarly, application of 75% RDF + Rhizobium + PSB to greengram produced positive net gain in phosphorus availability (5.5 kg/ha) followed by 100% RDF (4.8 kg/ha) and 75% RDF (4.4 kg/ha).

#### 3.5.3. Potassium Balance in Soil at the End of Cropping System

Data on potassium balance in soil is presented in Table 13. Integrated nutrient management practices with the rice–greengram system positively influenced the net balance of potassium in soil at the end of the system. Application of 50% RDF + 50% RDN through FYM to rice and 75% RDF + Rhizobium + PSB to greengram obtained the highest total potassium uptake (436.0 and 373.4 kg/ha).Net gain in potassium status in soil was maximum by application of 75% RDF + green manuring (26.9 kg/ha) followed by 50% RDF + 50% RDN through FYM (22.9 kg/ha) and 75% RDF + 25% RDN through FYM (22.5 kg/ha). The lowest gain was observed with 50% RDF + 25% RDN through treatment (16.6 kg/ha).

## 4. Discussion

Growth and development are a function of genetic, environmental factors and crop management practices that decide the overall crop growth in different stages of crop. Important growth attributes of rice that directs to higher yield are plant height, the number of tillers/m^2^, dry matter accumulation, and leaf area index. Integrated supply of organic and inorganic nutrients increased plant height of rice expeditiously up to 90 DAT. Further elongation was slower between 90 DAT and harvest due to the more demand of photosynthates at the sink after the reduction division stage to meet the reproductive requirement. With respect to nutrient management in aromatic rice, significantly taller plant height was noted with 50% RDF + 50% RDN through FYM in both the years which was at par with 75% RDF + green manuring and 75% RDF + 25% RDN through FYM. It might be due to the reason that integrated nutrient management practices increased in higher nutrient availability, resulting in increased conversion of carbohydrates to protein, which enhances meristematic cellular activity like cell division and cell elongation expressing morphologically in terms of increasing measured variables—such as plant height—ultimately increasing higher dry matter accumulation [23,24,25]. Similar results were also obtained by Asewar et al. [26], Sudha and Chandini [27] and Usman et al. [28], who observed increasing growth attributes with FYM + chemical fertiliser applied plots. Bellakki et al. [29] also opined that the higher performance of organic N as FYM or green manure might be due to a reduction in loss of N by fixation of NH^4+^ ion with humus present in FYM and timely availability of nitrogen to rice which ultimately enhanced the plant height.

Tiller production is the result of auxiliary bud expansion which is related to the nutritional requirement of the culm because the culm supplies carbohydrates and nutrients during the early stages which were enhanced by the application of nitrogen [30]. With the advancement in crop age, the number of tillers/m^2^ increased up to the stage of 90 DAT, and thereafter reduced. The reduction in the number of tillers after 90 days resulted from the senescence phase for which the secondary and tertiary tillers die. During the flowering stage, the leaf area index of aromatic rice was higher, after which point it declined until harvest because of the sufficient supply of nutrients to the plant and the potential of the plant for its absorption [31]. The combined application of organic manure and chemical fertilisers enhanced the production of carbohydrates which might have resulted in leaf expansion which in turn increased leaf area duration. These results corroborate the findings of Singh et al. [32] and Moe et al. [33].

Dry matter accumulation of aromatic rice increased progressively up to harvest and the enhancement was significant during flowering to harvest because, after heading and flowering, the increase in ear weight becomes noticeable. Significantly higher dry matter accumulation (770.4 and 757.1 g/m^2^) at harvest were found due to the application of 50% RDF + 50% RDN through FYM being at par with 75% RDF + green manuring and 75% RDF + 25% RDN through FYM. The factors for improving dry matter accumulation might be pertained because of the blending effect of organic nutrients on inorganic fertilisers which slowly and continuously release nutrients from the organic source at different growth stages and reduces nitrogen loss which prolongs the availability of N synchronizing with assimilation and absorption pattern of rice [34]. The lowest value was recorded from the application of 50% RDF + 50% RDN through FYM due to the sub-optimal application of organic manure and chemical fertiliser. Singh et al. [35] and Sudhagar Rao et al. [36] also recorded higher dry matter accumulation by combining application of organic manure and chemical fertiliser.The above-mentioned results clearly indicate the superior effect of integrated nutrient management over sole applications of inorganics.

### 4.1. Effects on Yield Attributes and Yield of Rice

Grain yield and straw yield are the final outcomes of a crop which generally relies on the development of yield attributes. An enhancement in growth characters because of the increased uptake of nitrogen and translocation of assimilates from source to sink increased most of the yield attributing characteristics.

Effective translocation of photosynthates to the sink probably favoured the cellular activities at the time of formation of panicles and development that led to sound filling of grains and increase in-ear bearing tillers/m^2^, ultimately leading to the highest number of filled grains/panicle. The proper partitioning might have occurred from source to sink and, as a result, the panicle length and lowest sterility percentage could have improved. The variation in 1000 grain weight might be due to differences in length and breadth of grain by application of FYM and green manure that are partly controlled by the genetic makeup of variety. These results are also supported by Yang et al. [37], Ahsan et al. [38], Mondal et al. [39], and Alagappan and Venkitaswamy [40].

Beneficial effects of FYM alone or in combination with fertilisers were earlier observed by Kumar et al. [41]. Higher grain productivity was recorded with inorganic fertilisers in combination with FYM or green manure might be because of greater availability and uptake of macro and micro-nutrients and active participation in carbon assimilation, photosynthesis, starch formation, sugar and protein translocation, water entry to the root of plants, etc. It also escalates the differentiation process from the somatic to the reproductive phase, leading to higher grain and straw yield of aromatic rice. Sumon et al. [42] and Sharma et al. [43] also opined that INM involving FYM and green manuring is imminent for sustainably increasing rice yield. An increase in rice grain yield due to green manure incorporation might be attributed to the release of nutrients to soils slowly for a longer period after decomposition, resulting in better yield attributes. The lowest grain and straw yield of aromatic rice was from the treatment receiving 50% RDF + 25% RDN through FYM due to the imbalance application of fertilisers. An increase in straw yield with INM treatments might be partly attributed to its direct influence on dry matter production of vegetative parts and indirectly through enhanced morphological parameters of growth. These results are confirmatory with Sharma et al. [43], Bhatt et al. [44], and Apon et al. [45]. Highest yield parameters such as the number of panicles m^−2^ (120.0), test weight (30.5 g), grain yield (3140 kg ha^−1^), and straw yield (8888 kg ha^−1^) were obtained due to the application of 75% RDF + 5 t FYM ha^−1^ despite the least values derived from that of sole application of 10 t FYM ha^−1^ [45].

Thus, the replacement of 50% chemical fertiliser with organic sources, viz., FYM provided an opportunity to harness the benefit of INM practices in aromatic rice.

### 4.2. Effect on Growth Attributes of Greengram

Growth attributes at different stages of greengram indicated that plant height and dry matter accumulation increased with the advancement of crop age and reached the peak at maturity during both years. Residual treatment of 50% RDF + 50% RDN through FYM to rice recorded significant taller plants and dry matter at harvest, the number of trifoliate leaves and LAI at 60 DAS in greengram; whereas, application of 75% RDF + *Rhizobium* + PSB to greengram increased all the growth parameters when compared with the application of 75% RDF and 100% RDF only. Farmyard manure is highly persistent bulky organic manure with a wider C:N ratio which results in slower decomposition. Therefore, nutrients from organic sources have not been fully exploited by the rice crop in the first crop season and possibly utilised by the following greengram crop. The slower release of nutrients for a longer duration due to mineralisation of undecomposed FYM favoured suitable microclimate by enhancing soil organic matter content; thus reducing bulk density compaction of soils. As a result, the plant gets an appropriate growing condition which facilitates better growth processes of greengram. Additionally, dual inoculation of *Rhizobium* and PSB to greengram helps the crop by providing atmospheric nitrogen for nitrogen fixation and supplying the insoluble phosphorus into a soluble form. The poor growth attributes of greengram under 75% RDF treatment were noted due to more intra-species competitiveness for infliction of available native soil nutrients, which results in lesser plant height, the number of leaves, and dry matter accumulation. These findings are closely related to the results of Bahadur and Tiwari [46], Armin et al. [47], and Meena et al. [48].

### 4.3. Effects of Residual Nutrients of Rice and Nutrient Management in Greengram on Yield Attributes and Yield of Greengram

Most of the yield attributing characters of greengram—viz., the number of pods plant^−1^, seeds pod^−1^, and pod length—were significantly influenced due to residual effect of INM applied to rice, as well as greengram. Due to a wider C:N ratio, the persistent material present in organic manures requires more time for decomposition so that almost 25% to 33% of nitrogen and a small fraction of phosphorus and potassium in FYM might be available to succeeding greengram crop. Significantly higher pods/plant, seeds/pod, pod length, and test weight were recorded due to residual treatment receiving 50% RDF + 50% RDN through FYM to rice with the application of 75% RDF + *Rhizobium* + PSB to greengram. Efficient utilisation of nitrogen generated by mineralisation from carrying over FYM and fertiliser N would have increased the availability of N throughout the growth period and thereby increased the assimilation of photosynthates which in turn transfers photosynthates from source to sink, leading to higher yield attributes of rice fallow greengram. Patel et al. [49] and Meena et al. [50] also reported similar results due to the combined application of chemical fertiliser with *rhizobium* and PSB inoculation.

Similarly, pooled data suggested that higher seed yield (806 and 697 kg/ha) and stover yield (1957 and 1767 kg/ha) were recorded by the residual treatment of 50% RDF + 50% RDN through FYM with 75% RDF + *Rhizobium* + PSB to greengram, respectively. It could be due to synergistic and cumulative carry-over effect of FYM addition which supplies nutrients on the one hand as well as a propensity to ameliorate the physicochemical and biological condition of soils, helping to achieve sustainable productivity of greengram crop for the long run under rainfed conditions. Synergism in *Rhizobium* and PSB inoculation to greengram might have also resulted in better yield. The increase in straw yield was probably because of high nitrogen availability to the plants from an optimal combined source of inorganics and organics. An enhancement in yield due to biofertiliser inoculation might not be only due to nitrogen fixation or phosphate solubilisation, but because of various factors—such as the release of growth promoters, plant-pathogen control, and accretion of beneficial organisms in the rhizosphere. A similar result was suggested by Parihar [51] that FYM being store-house of both macro-and micronutrients which might have enhanced the metabolic process vis-à-vis enlarged source and sink capacity, which finally enhanced the grain and straw yield. The lowest seed yield was obtained in 75% RDF alone treatment which registered 537 kg/ha. This might be due to decreased growth in terms of biomass accumulation during vegetative phases leading to decreased bearing capacity and could not get the required quantity of nutrients matching its demand which ultimately decreased the seed yield. The results are in the line with the findings of Singh et al. [52]. A similar enhancement in yield attributes of greengram because of INM adopted to previous crops has been reported by Krishna Kumar et al. [53], Alagappan and Venkitaswamy [40], and Pandey et al. [54]. Alagappan and Venkitaswamy [39] opined that residual greengram recorded higher grain yield (642 kg/ha and 698 kg/ha) for two subsequent seasons with INM practice (RDF + green manure at 6.25 t/ha) followed by RDF treatment in preceding rice (437 kg/ha and 502 kg/ha). Among the organics, higher seed yield was recorded with TRRI practice (642 kg ha^−1^ and 698 kg/ha) followed by 100% RDN through green manure in preceding rice (410 kg ha^−1^ and 476 kg/ha) in both the years of study. Armin et al. [47] reported that the highest number of pods plant^−1^ (25.1), number of seeds pod^−1^ (14.9), number of seeds plant^−1^ (319.6), test weight (41.9 g), seed yield plant^−1^ (15.2 g), and seed yield (1156.2 kg/ha) in greengram recorded from 100% RDF + vermicompost. There is a little variation in the harvest index of greengram due to the combined application of organics and inorganics. The reason is that as the application rate of both the inorganic and organic sources of nutrients increase both the grain yield and the above-ground biomass yield directly, it exerts little or no change on the ratio of the grain yield to the above-ground biomass yield.

### 4.4. Nutrient Balance in the System

A significant increase of available nitrogen content in soil was found due to the applications of 75% RDF + green manuring to rice and 75% RDF + Rhizobium + PSB to greengram respectively over their initial status. The increase in available nitrogen content in soil due to the application of green manure (*dhaincha*) and FYM might be due to mineralisation of nitrogen and soil microbe multiplication, which converts organically bound nitrogen into an available form. Additionally, dual inoculation of Rhizobium and PSB converts atmospheric nitrogen to the available form of nitrogen through nitrogen fixation. Similarly, higher available phosphorus and potassium in soil were found due to the application of 75% RDF + green manuring to rice and 75% RDF + *Rhizobium* + PSB to greengram. This might be due to the combined use of organic and inorganic sources of nutrients which, in turn, could attribute better synchrony of nutrient availability to the rice crop resulting in better biomass production and higher nitrogen use efficiency. Similar findings are also reported by Mwale et al. [55] and Balasubramaniam et al. [56]. INM practices were found to enhance the microbial activity which in turn stimulated the conversion of unavailable nutrients into available form through improving the physicochemical properties of the soil [57]. The build-up in available P with the conjoint use of fertilisers and organics could be ascribed to the release of organic acids during decomposition, which in turn helped in releasing native phosphorus through solubilizing action of these acids. Additionally, organic matter forms a coating on sesquioxides and makes them inactive and thus reduces the phosphate fixing capacity of the soil, which ultimately helps in the release of ample quantity of plant-available P as observed by Mohandas and Appavu [58].

## 5. Conclusions

The present study revealed that nutrient management in aromatic rice produced significantly taller plants with 50% RDF + 50% RDN through FYM, which was at par with 75% RDF + green manuring and 75% RDF + 25% RDN through FYM. The above treatment also resulted in maximum values in terms of, more dry matter, and a greater number of tillers m^−2^, grain, and straw yields. The INM enhanced the production of carbohydrates which might have increased leaf expansion and leaf area duration. The residual effect of 50% RDF + 50% RDN through FYM applied to rice recorded significantly taller plants and dry matter at harvest, the number of trifoliate leaves and LAI at 60 DAS, yield attributes, seed, and stover yields in greengram; whereas maximum values for above parameters were recorded with the application of 75% RDF + *Rhizobium* + PSB to the greengram. The results concluded that the productivity of the short grain aromatic rice–greengram system in the east and south-eastern coastal plain agro-climatic zone of Odisha can be alleviated by the adoption of INM. Integration of 50% RDF (60-30-30 N–P_2_O_5_-K_2_O kg/ha) coupled with 50% RDN through FYM to aromatic rice improved the growth productivity of aromatic rice; however, residual application of 50% RDF coupled with 50% RDN through FYM to rice and application of 75% RDF + *Rhizobium* + PSB to greengram was recorded to perform the best. Furthermore, INM positively impacted the nutrient balance of the soil.

## Figures and Tables

**Table 1:** Chemical composition of the experimental site.

Composition	Value	Method Employed
Bulk density (g/cc)	1.55	Core sampler method [17]
Particle density (g/cc)	2.67	Pycnometer method [17]
pH (1:2.5::soil:water)	5.66	Glass electrode Beckman’s electronic pH metre [18]
Electrical conductivity (dS/m) (1:2.5::soil:water)	0.13	Conductivity metre [18]
Water holding capacity (%)	39.4	Keen Raczkowski box method [19]
Organic carbon (g/kg)	3.82	Walkley and Black method [18]
Available N (kg/ha)	199.3	Alkaline permanganate method [20]
Available P (kg/ha)	17.3	Bray’s-1 method [18]
Available K (kg/ha)	269.1	Neutral normal ammonium acetate method [18]
Iron (ppm)	140.2	DTPA method [21]
Manganese (ppm)	8.22	DTPA method [21]
Zinc (ppm)	0.59	DTPA method [21]
Copper (ppm)	1.19	DTPA method [21]

**Table 2:** Nutrient content of organic manures used in the experiment.

Organic Source	Year	N (%)	P (%)	K (%)
FYM	2017	0.63	0.36	0.62
	2018	0.58	0.40	0.55
Dhaincha (*Sesbania aculeata*)	2017	3.44	0.74	1.12
	2018	3.56	0.82	1.25

**Table 3:** Effect of integrated nutrient management on plant height of short grain aromatic rice at different growth stages.

Treatment	Plant Height (cm)							
	30 DAT		60 DAT		90 DAT		At Harvest	
	2017	2018	2017	2018	2017	2018	2017	2018
G1	43.7 ^bc^	45.5 ^b^	107.6 ^c^	109.9 ^c^	116.6 ^c^	119.3 ^cd^	119.2 ^c^	122.6 ^c^
G2	45.9 ^b^	47.9 ^b^	112.3 ^bc^	112.1 ^bc^	120.7 ^bc^	122.6 ^c^	122.8 ^bc^	124.0 ^c^
G3	53.0 ^a^	53.6 ^a^	123.3 ^a^	126.5 ^a^	132.0 ^a^	135.9 ^a^	135.4 ^a^	138.1 ^a^
G4	39.8 ^c^	40.4 ^c^	100.8 ^d^	95.3 ^d^	108.4 ^d^	108.2 ^e^	111.9 ^d^	111.0 ^d^
G5	51.3 ^a^	52.7 ^a^	116.4 ^b^	116.7 ^b^	124.9 ^b^	129.2 ^b^	127.9 ^b^	131.4 ^b^
G6	45.2 ^b^	45.9 ^b^	109.6 ^c^	107.2 ^c^	115.4 ^c^	113.5 ^de^	118.6 ^c^	116.2 ^d^
SE(m) ±	2.28	2.44	4.13	5.41	4.24	4.39	4.19	5.05
LSD (0.05)	7.18	7.70	13.02	17.06	13.36	13.82	13.20	15.90

G_1_: 100 % recommended dose of fertiliser (60-30-30 kg/ha of N-P_2_O_5_-K_2_O), G_2_: 75% RDF + 25% RDN through FYM, G_3_: 50% RDF + 50% RDN through FYM, G_4_: 50% RDF + 25% RDN through FYM, G_5_:75% RDF + green manuring and G_6_: 50% RDF + green manuring. Data were subjected to Tukey’s HSD test. SE, Standard errors and LSD, least significance difference at 5% probability level. Different superscript letters indicate significant differences between means.

**Table 4:** Effect of integrated nutrient management on tillers m^−2^ of rice at different growth stages.

Treatment	Tillers/m^2^					
	30 DAT		60 DAT		90 DAT	
	2017	2018	2017	2018	2017	2018
G1	76.3 ^c^	88.9 ^c^	251.8 ^d^	264.4 ^c^	306.4 ^d^	313.6 ^d^
G2	87.5 ^b^	100.6 ^b^	275.8 ^c^	286.7 ^b^	330.1 ^c^	336.6 ^c^
G3	99.1 ^a^	111.6 ^a^	304.0 ^a^	312.5 ^a^	359.3 ^a^	372.6 ^a^
G4	74.8 ^c^	87.9 ^c^	227.1 ^e^	239.0 ^d^	274.2 e	292.7 ^e^
G5	100.2 ^a^	106.6 ^ab^	289.7 ^b^	303.9 ^a^	341.9 ^b^	351.0 ^b^
G6	91.7 ^b^	104.4 ^b^	260.1 ^d^	274.2 ^c^	319.3 ^c^	323.9 ^d^
SE(m) ±	4.25	5.23	12.42	14.73	16.23	14.59
LSD (0.05)	13.38	16.46	39.13	46.41	51.15	45.97

For treatment details, Table 2 may be referred. SE, Standard errors and LSD, least significance difference at 5% probability level. Different superscript letters indicate significant differences between means.

**Table 5:** Effect of Integrated nutrient management on dry matter accumulation of short grain aromatic rice at different growth stages.

Treatments	Dry Matter Accumulation of Rice							
	30 DAT		60 DAT		90 DAT		Harvest	
	2017	2018	2017	2018	2017	2018	2017	2018
G1	132.6 ^d^	119.5 ^e^	290.5 ^d^	303.6 ^d^	545.4 ^d^	541.3 ^c^	698.4 ^d^	684.2 ^d^
G2	170.7 ^b^	160.5 ^c^	328.3 ^b^	319.3 ^c^	590.9 ^b^	579.9 ^b^	738.4 ^b^	729.7 ^b^
G3	181.3 ^a^	192.9 ^a^	374.8 ^a^	361.5 ^a^	617.6 ^a^	612.6 ^a^	770.4 ^a^	757.1 ^a^
G4	133.9 ^d^	139.7 ^d^	282.5 ^d^	289.6 ^e^	521.7 ^e^	510.0 ^d^	600.0 ^e^	583.9 ^e^
G5	173.1 ^ab^	175.7 ^b^	334.4 ^b^	341.7 ^b^	603.2 ^b^	608.6 ^a^	743.9 ^b^	750.7 ^a^
G6	159.1 ^c^	161.7 ^c^	308.2 ^c^	318.3 ^c^	558.4 ^c^	568.8 ^b^	716.6 ^c^	702.1 ^c^
SE(m) ±	8.60	7.04	13.51	14.13	19.25	21.42	26.03	23.66
LSD (0.05)	27.09	22.18	42.56	44.51	60.65	67.48	82.02	74.56

For treatment details, Table 2 may be referred. SE, Standard errors and LSD, least significance difference at 5% probability level. Different superscript letters indicate significant differences between means.

**Table 6:** Effect of integrated nutrient management on yield parameters of short grain aromatic rice.

Treatment	EBT		Filled Grains/Panicle		Grain Yield (kg/ha)		Straw Yield (kg/ha)		Harvest Index (%)	
	2017	2018	2017	2018	2017	2018	2017	2018	2017	2018
G1	268.8 ^d^	286.4 ^c^	133.8 ^d^	142.5 ^d^	2983 ^e^	3113 ^e^	4609 ^e^	4960 ^c^	39.3 ^bc^	38.5 ^cd^
G2	295.8 ^c^	302.6 ^b^	147.7 ^c^	151.0 ^c^	3346 ^c^	3328 ^c^	4881 ^c^	5111 ^b^	40.6 ^ab^	39.4 ^cd^
G3	315.7 ^a^	321.0 ^a^	178.6 ^a^	182.6 ^a^	3837 ^a^	3914 ^a^	5278 ^a^	5342 ^a^	42.1 ^a^	42.2 ^a^
G4	255.4 ^e^	260.4 ^d^	125.2 ^e^	132.5 ^e^	2580 ^f^	2697 ^f^	4297 ^f^	4499 ^e^	37.5 ^c^	37.4 ^d^
G5	305.4 ^b^	312.6 ^a^	160.2 ^b^	164.2 ^b^	3438 ^b^	3539 ^b^	4924 ^b^	4931 ^c^	41.1 ^ab^	41.8 ^ab^
G6	273.1 ^d^	290.6 c	138.6 ^d^	143.4 ^d^	3182 ^d^	3224 ^d^	4802 ^d^	4864 ^d^	39.8 ^abc^	39.8 ^bc^
SE(m) ±	10.68	11.01	5.42	6.43	143.0	142.6	144.0	130.0	0.68	0.63
LSD (0.05)	33.65	34.68	17.07	20.27	450.6	449.4	453.7	409.6	2.14	1.97

For treatment details, Table 2 may be referred. SE, Standard errors and LSD, least significance difference at 5% probability level. Different superscript letters indicate significant differences between means.

**Table 7:** Multiple correlations between yield parameters and grain yield of rice.

Parameters	Ear Bearing Tillers/m^2^	Panicle Length (cm)	Filled Grains/Panicle	Test Weight (g)	Grain Yield (kg/ha)
Ear bearing tillers/m^2^	1				
Panicle length	0.84	1			
Filled grains/panicle	0.71	0.67	1		
Test weight	0.59	0.65	0.87	1	
Grain yield	0.79	0.76	0.82	0.77	1

**Table 8:** Effect on Integrated nutrient management on plant height (cm) of rabi greengram.

Treatment	Plant Height							
	30 DAS		45 DAS		60 DAS		Harvest	
	2017	2018	2017	2018	2017	2018	2017	2018
Nutrient management in rice								
G1	16.5 ^d^	18.7 ^c^	26.9 ^c^	28.1 ^c^	31.1 ^b^	31.8 ^b^	31.9 ^bc^	32.5 ^bc^
G2	20.3 ^c^	20.9 ^b^	29.9 ^b^	30.1 ^b^	32.3 ^b^	33.1 ^b^	33.2 ^b^	34.3 ^b^
G3	24.0 ^a^	23.8 ^a^	32.0 ^a^	32.4 ^a^	35.4 ^a^	36.2 ^a^	36.8 ^a^	37.8 ^a^
G4	15.8 ^d^	17.1 ^d^	24.8 ^d^	24.9 ^d^	28.1 ^c^	28.3 ^c^	29.1 ^c^	30.1 ^c^
G5	22.4 ^b^	22.9 ^a^	32.3 ^a^	31.3 ^ab^	35.0 ^a^	35.1 ^a^	36.0 ^a^	36.8 ^a^
G6	19.6 ^c^	20.3 ^b^	28.2 ^c^	28.6 ^c^	31.9 ^b^	32.4 ^b^	32.8 ^b^	33.7 ^b^
SE(m) ±	0.24	0.24	0.27	0.23	0.72	0.34	0.73	0.72
LSD (0.05)	0.75	0.75	0.84	0.72	2.28	1.08	2.29	2.27
Nutrient management in greengram								
P1	20.1 ^a^	20.8 ^b^	29.3 ^a^	29.8 ^b^	32.6 ^a^	33.1 ^b^	33.6 ^a^	34.6 ^a^
P2	18.2 ^b^	19.5 ^c^	27.9 ^b^	26.9 ^c^	30.5 ^b^	31.3 ^c^	31.3 ^b^	32.2 ^b^
P3	21.1 ^a^	21.6 ^a^	29.9 ^a^	31.0 ^a^	33.8 ^a^	34.1 ^a^	34.9 ^a^	35.8 ^a^
SE(m) ±	0.27	0.15	0.26	0.17	0.47	0.12	0.45	0.47
LSD (0.05)	0.78	0.43	0.76	0.49	1.38	0.35	1.33	1.36

For treatment details, Table 2 may be referred. SE, Standard errors and LSD, least significance difference at 5% probability level. Different superscript letters indicate significant differences between means.

**Table 9:** Effect on integrated nutrient management on dry matter accumulation (g/plant) of *rabi* greengram.

Treatment	Dry Matter Accumulation (g/Plant)							
	30 DAS		45 DAS		60 DAS		Harvest	
	2017	2018	2017	2018	2017	2018	2017	2018
Nutrient management in rice								
G1	2.7 ^c^	2.9 ^c^	6.6 ^d^	6.8 ^d^	10.4 ^d^	10.7 ^d^	11.6 ^d^	11.8 ^d^
G2	3.7 ^b^	3.7 ^b^	9.2 ^b^	9.7 ^b^	12.3 ^c^	12.5 ^c^	13.6 ^c^	13.7 ^c^
G3	4.9 ^a^	5.0 ^a^	10.9 ^a^	11.4 ^a^	14.6 ^a^	14.9 ^a^	15.9 ^a^	16.3 ^a^
G4	2.6 ^c^	2.6 ^c^	5.7 ^d^	5.9 ^d^	7.8 ^e^	7.9 ^e^	8.9 ^e^	9.1 ^e^
G5	4.4 ^a^	4.5 ^a^	10.3 ^a^	10.9 ^a^	13.3 ^b^	13.7 ^b^	14.6 ^b^	15.0 ^b^
G6	2.8 ^c^	2.8 ^c^	7.8 ^c^	8.0 ^c^	11.4 ^c^	11.8 ^c^	12.9 ^c^	13.3 ^c^
SE(m)±	0.06	0.05	0.13	0.13	0.11	0.13	0.10	0.12
LSD (0.05)	0.20	0.16	0.40	0.42	0.34	0.40	0.33	0.38
Nutrient management in greengram								
P1	3.6 ^b^	3.6 ^b^	8.5 ^b^	8.8 ^b^	11.6 ^b^	11.8 ^b^	12.9 ^b^	13.1 ^b^
P2	3.0 ^c^	3.0 ^c^	7.4 ^c^	8.0 ^c^	10.5 ^c^	10.8 ^c^	11.7 ^c^	11.9 ^c^
P3	4.0 ^a^	4.1 ^a^	9.3 ^a^	9.5 ^a^	12.8 ^a^	13.1 ^a^	14.2 ^a^	14.6 ^a^
SE(m)±	0.02	0.03	0.06	0.11	0.06	0.07	0.07	0.08
LSD (0.05)	0.06	0.10	0.19	0.31	0.19	0.19	0.20	0.23

For treatment details, Table 2 may be referred. SE, standard errors and LSD, least significance difference at 5% probability level. Different superscript letters indicate significant differences between means.

**Table 10:** Effect of integrated nutrient management on yield parameters of *rabi* greengram.

Treatment	Pods/Plant		Seeds/Pod		Pod Length (cm)		Seed Yield (kg/ha)		Stover Yield (kg/ha)		Harvest Index (%)	
	2017	2018	2017	2018	2017	2018	2017	2018	2017	2018	2017	2018
G1	11.4 ^c^	12.5 ^c^	7.0 ^c^	6.6 ^c^	5.4 ^c^	6.2 ^b^	511 ^d^	539 ^e^	1477 ^e^	1411 ^e^	25.6 ^bc^	27.6 ^bc^
G2	13.3 ^b^	14.7 ^b^	8.7 ^b^	9.3 ^b^	6.3 ^bc^	6.4 ^b^	611 ^c^	629 ^c^	1685 ^c^	1612 ^c^	26.6 ^ab^	28.0 ^abc^
G3	16.1 ^a^	17.0 ^a^	10.3 ^a^	11.2 ^a^	8.26 ^a^	8.30 ^a^	798 ^a^	815 ^a^	1993 ^a^	1922 ^a^	28.5 ^a^	29.7 ^a^
G4	10.6 ^c^	11.9 ^c^	6.5 ^c^	6.3 ^c^	5.34 ^c^	5.7 ^b^	479 ^e^	495 ^f^	1462 ^e^	1324 ^f^	24.5 ^c^	27.1 ^bc^
G5	15.2 ^a^	16.4 ^a^	10.6 ^a^	10.8 ^a^	7.90 ^a^	8.2 ^a^	702 ^b^	733 ^b^	1816 ^b^	1811 ^b^	27.8 ^a^	28.7 ^ab^
G6	12.9 ^b^	14.2 ^b^	8.4 ^b^	9.9 ^b^	6.44 ^b^	6.6 ^b^	521 ^d^	558 ^d^	1570 ^d^	1563 ^d^	24.9 ^bc^	26.5 ^c^
SE(m) ±	0.19	0.19	0.19	0.05	0.12	0.27	23.52	21.62	44.37	33.71	0.45	0.49
LSD (0.05)	0.61	0.60	0.58	0.15	0.37	0.86	74.11	68.14	139.8	106.21	1.43	1.56
P1	13.1 ^b^	14.3 ^b^	8.7 ^b^	9.0 ^b^	6.7 ^a^	7.0 ^a^	606 ^b^	620 ^b^	1685 ^b^	1603 ^b^	26.3 ^b^	27.7 ^b^
P2	12.4 ^c^	13.5 ^c^	7.7 ^c^	8.7 ^b^	6.0 ^b^	6.4 ^b^	520 ^c^	554 ^c^	1532 ^c^	1468 ^c^	25.1 ^c^	27.3 ^c^
P3	14.2 ^a^	15.6 ^a^	9.4 ^a^	9.4 ^a^	7.1 ^a^	7.3 ^a^	684 ^a^	710 ^a^	1784 ^a^	1749 ^a^	27.6 ^a^	28.8 ^a^
SE(m) ±	0.11	0.12	0.08	0.03	0.08	0.07	7.08	8.34	23.37	16.36	0.29	0.32
LSD (0.05)	0.31	0.34	0.24	0.09	0.23	0.22	20.68	24.33	68.20	47.74	0.85	0.93

For treatment details, Table 2 may be referred. SE, standard errors and LSD, least significance difference at 5% probability level. Different superscript letters indicate significant differences between means.

**Table 11:** Nitrogen balance in the soil at the end of cropping system.

Treatment	Initial Fertility (kg/ha)	N Added (kg/ha)	N Uptake (kg ha^−1^)	Computed Balance (kg ha^−1^)	Actual Fertility after Harvest (kg ha^−1^)	Net Gain/Loss (kg ha^−1^)
Nutrient management in rice						
G1	199.3	153.3	227.4	−74.1	210.5	11.2
G2	199.3	153.3	264.1	−110.8	218.8	19.5
G3	199.3	153.3	332	−178.7	221.2	21.9
G4	199.3	153.3	201.5	−48.2	200.7	1.4
G5	199.3	420.7	288.9	131.8	225.7	26.4
G6	199.3	384.7	240.4	144.3	213.6	14.3
Nutrient management in greengram						
P1	199.3	243.1	257.1	−14.0	215	15.7
P2	199.3	233.1	243.1	−10.0	213.1	13.8
P3	199.3	233.1	271.9	−38.8	217.1	17.8

For treatment details, Table 2 may be referred.

**Table 12:** Phosphorus balance in soil at the end of cropping system.

Treatment	Initial Fertility (kg/ha)	P Added (kg/ha)	P Uptake (kg/ha)	Computed Balance (kg/ha)	Actual Fertility after Harvest (kg/ha)	Net Gain/Loss (kg/ha)
Nutrient management in rice						
G1	17.3	126.7	37.7	89.0	20.4	3.1
G2	17.3	130.6	45.1	85.5	21.5	4.2
G3	17.3	135.6	57.4	78.2	24.5	7.2
G4	17.3	115.6	30.7	84.9	19.6	2.3
G5	17.3	176.6	53.1	123.5	26.2	8.9
G6	17.3	161.6	41.1	120.5	20.9	3.6
Nutrient management in greengram						
P1	17.3	154.4	44.3	110.1	22.1	4.8
P2	17.3	134.4	42.1	92.3	21.7	4.4
P3	17.3	134.4	46.1	88.3	22.8	5.5

For treatment details, Table 2 may be referred.

**Table 13:** Potassium balance in soil at the end of cropping system.

Treatment	Initial Fertility (kg/ha)	K Added (kg/ha)	K Uptake (kg/ha)	Computed Balance (kg/ha)	Actual Fertility after Harvest (kg/ha)	Net Gain/Loss (kg/ha)
Nutrient management in rice						
G1	269.1	126.7	332.8	−206.1	288.3	19.2
G2	269.1	140.6	377.6	−237.0	291.6	22.5
G3	269.1	156.1	436.0	−279.9	292.0	22.9
G4	269.1	125.6	304.9	−179.3	285.7	16.6
G5	269.1	210.2	392.0	−181.8	296.0	26.9
G6	269.1	195.2	350.2	−155.0	290.8	21.7
Nutrient management in greengram						
P1	269.1	172.4	365.9	−193.5	291.3	22.2
P2	269.1	152.4	357.3	−204.9	289.1	20.0
P3	269.1	152.4	373.4	−221.0	291.8	22.7

For treatment details, Table 2 may be referred.

## Data Availability

Data recorded in the current study are available in all Tables and Figures of the manuscript.
